# Treating symptomatic flexible flatfoot deformities. a novel technique: comparison of uc berkeley laboratory foot orthosis with and without kinesio taping in juvenil athletes

**DOI:** 10.1007/s00264-024-06205-5

**Published:** 2024-05-08

**Authors:** Cem Sever, Eşref Terzi, Akif Kurtan, Sami Sökücü, Aybars Kıvrak, Kubilay Beng

**Affiliations:** 1 Department of Orthopedics and Traumatology, Bahçeşehir University Medical Park Goztepe Hospital, İstanbul, Türkiye; 2 Department of Orthopedics and Traumatology, Avrupa Hospital, Şehitler Bulvarı Avrupa Hastanesi Cukurova, Adana, Türkiye; 3 Department of Orthopedics and Traumatology, Medical Park Goztepe Hospital, İstanbul, Türkiye; 4 Department of Orthopedics and Traumatology, İstanbul Aydın University Medical Park Florya Hospital, İstanbul, Türkiye

**Keywords:** Flatfoot deformity, Foot orthosis, Kinesio taping, Pes planus, UCBL

## Abstract

**Purpose:**

Symptomatic flexible pes planus (SFPP) can cause pain and discomfort when walking or engaging in sportive activities in children and adolescents. SFPP can be treated conservatively with foot orthoses, such as the University of California Berkeley Laboratory (UCBL) foot orthosis, which can improve foot function and reduce pain. Kinesio Tape (KT) has also been used as an adjunct to foot orthoses in the treatment of pes planus. This study aims to compare the effectiveness of the UCBL foot orthosis with and without KT in the treatment of SFPP among amateur juvenile and adolescent athletes.

**Methods:**

Fifty patients with SFPP were included in the study. In 27 patients UCBL foot orthosis with KT (group 1) was used whereas in 23 UCBL (group 2) was preferred only. The patients were evaluated with AOFAS and radiological measurements.

**Results:**

The mean follow-up period was 28.6 ± 4.3(26) months. At the final follow-up AOFAS of group 1 was significantly higher than group 2. In group 2, 12 patients (%52,17) had pressure sores that caused superficial dermabrasion. Lateral TFMAs and talocalcaneal angle in group 1 was significantly better than group 2.

**Conclusions:**

This study attempted to determine if using KT with the UCBL foot orthosis was beneficial to the treatment of SFPP compared to simply wearing the orthosis. Our results suggest that KT is effective in reducing pronation and improving the AOFAS score. The use of UCBL with KT seems to be preferable in children and adolescents with SFPP since it is associated with a lower rate of complication, a higher degree of patient compliance and faster improvement in the radiological and clinical findings, compared to the use of the UCBL orthosis alone.

## Background

Symptomatic flexible pes planus (SFPP) is a dynamic dysfunction that can lead to serious complications such as limited mobility, calf and foot pain, and reduced quality of life [1, 2]. Hindfoot valgus deviation and increased talar tilt are primarily associated with SFPP [3, 4]. Talar subluxation alters the kinetic chain and results in shortening of the Achilles tendon and limits the function of the posterior tibial tendon [5]. Reduced longitudinal arch height and increased forefoot abduction with rearfoot eversion in patients with severe flat foot trigger symptoms that lead to changes in the mechanical axis of the extremities.

Especially for children aged three to six years, muscle training and exercise are as effective as orthotics and surgery [2, 6, 7]. Foot orthotics and shoe modifications, soft tissue reconstruction, calcaneal osteotomy, and arthrodesis are options for the treatment of SFPP [8]. Structural deformities and induced changes in foot pressure distribution and other anatomical regions are taken into account when selecting treatment [9–11]. Techniques that attempt to correct hyperpronation include prescription orthosis and banding techniques [12]. However, prolonged use of these orthoses can cause some pressure points on bony prominences and can also lead to non-adherence to treatment.

The use of Kinesio Taping (KT) as a complementary treatment in orthopedic pathology and sports medicine and has increased in recent years [12].KT is similar in thickness to the epidermis and can be stretched longitudinally by 30% to 40% of its original length. Low-stain taping and high-stain taping techniques have also been described to correct foot pronation [13, 14]. However, to our knowledge, the use of KT in SFPP has not been studied.

Our hypothesis is: "We can avoid complications of foot orthoses such as pressure sores of the talus and medial/lateral malleolus and increase the compliance of the patients and the effectiveness of deformity correction through the use of UCBL with KT". In this retrospective study, we evaluated radiographic and clinical outcomes and assessed the effectiveness of the orthosis with and without KT.

## Material and methods

### Material

For this study, we declare that the informed consent is obtained. We state that all rights of all subjects are protected**.** This retrospective comparative study was approved by the Ethics Committee of Istanbul Medical University (Approval No. 311 Ethics Committee No.: 10840098–604.01.01-E.7684) Sixty-one patients with a definitive diagnosis of SFPP based on radiographic and clinical admitted to our clinic in May 2015 and June 2017.

Patients who: (1) have symptoms that worsen when standing, walking, or running for long periods, (2) have a significant deformity of the longitudinal arch or shoe load, or complain of calf pain, (3) have not had any foot surgery and (4) willing to participate in the study. Subjects with (1) tibiotalar or subtalar joint stiffness or (3) a history of an allergic reaction to KT or plastazote were excluded from the study. Eight patients lost to follow-up and three patients with allergic reactions in group 1 were excluded from the study. A total of 50 SFPP patients (mean age 8.9 years; range 6.5 to 13.1 years) totalling 100 feet participated in the study. Twenty-seven patients (group 1; 13 males, 14 females; mean age 8.3 months; range 7.1 to 13.1 years) used UCBL foot orthoses with KT, while the remaining 23 (group 2; 13 males, 10 females; mean age 9.1 years, range 6.5 to 11.8 years) UCBL was the first choice without KT (Table 1). The average follow-up time was 28.6 ± 4.3(26) months. According to available data, there were no statistically significant differences in age and sex between the two groups (p = 0.555 and 0.861, respectively) (Table 1).

**Table 1:** Evaluation of demographic data

		Participants		P
		Group 1 (n = 27)	Group 2 (n = 23)	
Gender; n(%)	Male	13 (48,1)	13 (56,5)	a0,555
	Female	14 (51,9)	10 (43,5)	
Age (years)	Mean ± SD	9.2 ± 2.9	8.2 ± 1.7	b0,861
	Min–Max (Median)	7.1–13.1 (8.3)	6.5–11.8 (9.1)	

aPearson chi-spuare Test bMann- Whitney U Test

### Methods

Functional health of the foot and ankle were recorded using the AOFAS every six weeks and radiological evaluations by measuring talocalcaneal (TCA) and talo-first metatarsal (TFMA), calcaneal pitch (CPA) and talonavicular (TNA) angles in every six months as described and validated in several studies [15–17]. The x-rays of the patients were analyzed before treatment and at the sixth and 12th month follow-ups. The methods described by Gould, Perry et al. and Sangeorzan et al. were used to measure the axes of the calcaneus, talus, and first metatarsal [18–20].

**Fig. 1:**
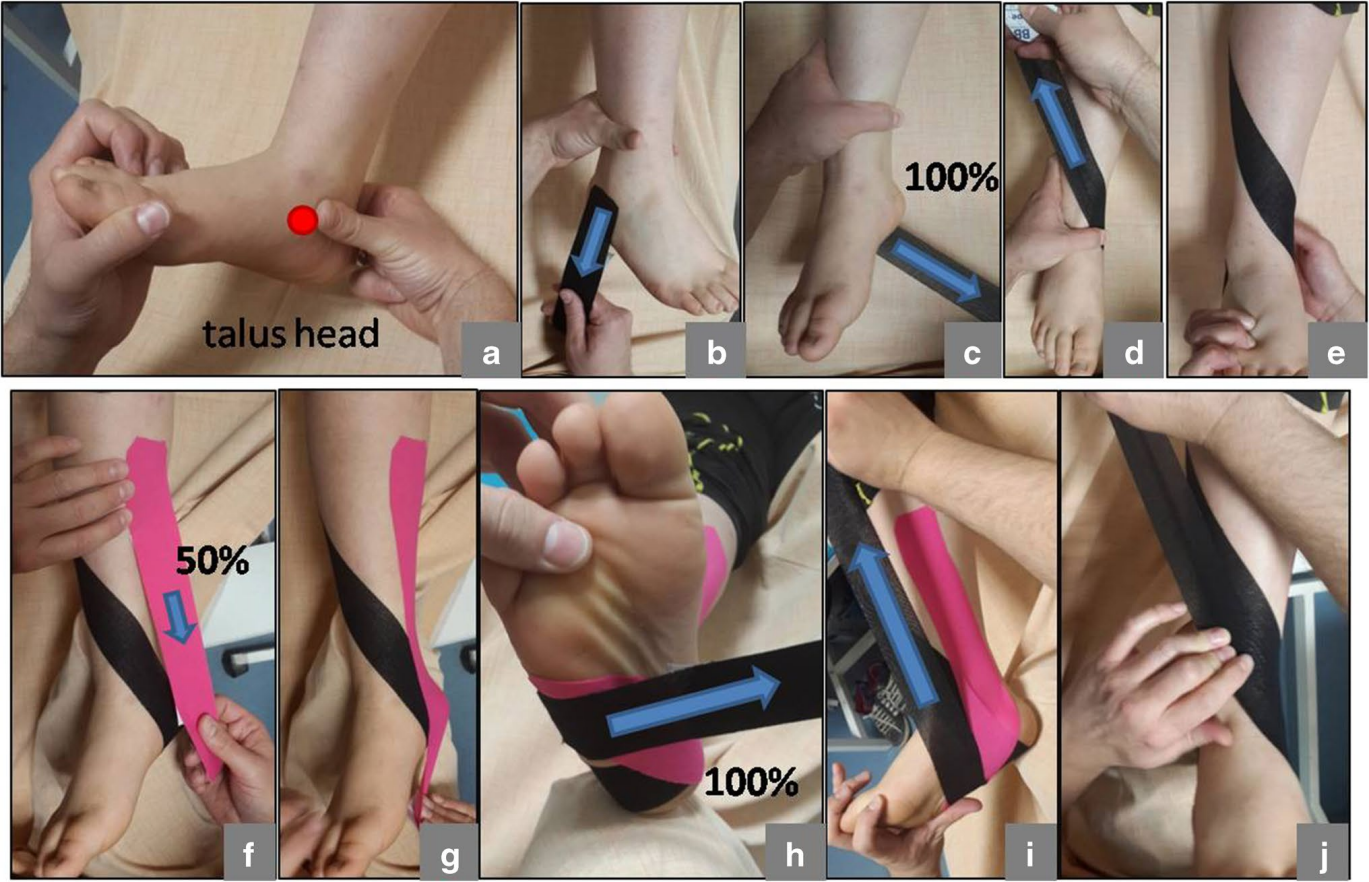
Application technique. Talar head is reduced and calcaneovalgus corrected (a). A standard 5-cm BBtape© was used. The first strip, in varying lengths according to the patient’s foot size, was applied from the fibula (lateral malleolus) (b), around the calcaneus, with a 100% stretch, up to the middle third of the medial tibia (c and d). The strip was applied to rear foot in a supinated position (d and e). The second strip was applied on the projection of the tibialis posterior muscle and the tendon on the skin, starting from the origin of the muscle with a 50% stretch, up to the insertion of the tendon on the navicular bone (f and g). The third strip was applied from the longitudinal arch with a 100% stretch to the middle third of the tibia, which lied parallel to the first strip, trying to restore the flattened footpad (h,i and j). After application, the instructor warmed the KT by rubbing his hand several times from the starting point to the end point in order to maximize its adhesion

KT application technique is shown in Fig. 1. All KT applications were applied by the first author. The strips remained on the patient for three days. On the fourth day, bare skin for self-sanitization. KT was applied again after a one-day interval. All patients received custom-made UCBL foot orthoses, a thermoplastic in-shoe orthosis designed to limit hindfoot motion and correct talar inclination. All orthoses were custom-made and all molds were taken by the first author (CS) during the correction of talar tilt and calcaneal valgus. To avoid pressure sores, the mold is coated with plastazote and reinforced with medial support to prevent longitudinal arch collapse. All patients in both groups used the UCBL orthosis for at least eight hours per day. Foot pronation was assessed immediately after taping, then at each follow-up visit and the end of treatment. When assessing pediatric participants, pay special attention to being in the position they feel most comfortable with.

Criteria for discontinuation of treatment were; symptom resolution, normalization of the talo-calcaneal angle and improvement of the AOFAS score. Patients are assessed and evaluated at each visit for orthosis-related pressure sores.

### Assessment

AOFAS scores and radiological measurements of the patients were performed by the second author. Radiological measurements were performed as previously described in the literature [15–17]. Radiological measurements were performed using standard DICOM viewing software (RadiAnt Dicom Viewer Version 2023.1). The author who performed the radiological evaluations was blinded to the treatment.

 NCSS 2007 software (NCSS, LLC, Kaysville, UT, USA) was used for statistical evaluation. Descriptive statistical methods (means, standard deviations, medians, frequencies, and ratios) commonly used to compare quantitative data and distributions of independent variables were used in the data analysis. Independent samples test (Student's t-test). Results were evaluated with 95% confidence intervals and a significance level of p < 0.05.

## Results

### Outcome measures

There was no significant difference in the AOFAS score between the two groups before treatment (p > 0.05). On the other hand, the AOFAS scores before and after treatment were significantly improved in both groups (p = 0.001). The forefoot and midfoot AOFAS scores were significantly improved from 58.00 ± 5.83 to 78.00 ± 5.83 (p = 0.001) in group 1 and from 56.00 ± 5.69 to 64.00 ± 5.69 (p = 0.024) in group 2 (Table 2). At the final follow-up, the AOFAS of group 1 was significantly higher than that of group 2.

**Table 2:** Evaluation of AOFAS values

AOFAS	Participants		P
	Group 1 (n = 27) Ort ± SD	Group 2 (n = 23) Ort ± SD	
Pre-treatment	58,00 ± 5,83	56,00 ± 5,69	a0,228
6th week	62,00 ± 5,83	58,00 ± 5,69	0,018*
12th week	66,00 ± 5,83	58,00 ± 5,69	0,001**
18th week	68,00 ± 5,83	60,00 ± 5,69	0,001**
24th week	68,00 ± 5,83	60,00 ± 5,69	0,001**
30th week	70,00 ± 5,83	60,00 ± 5,69	0,001**
36th week	70,00 ± 5,83	62,00 ± 5,69	0,001**
42nd week	72,00 ± 5,83	62,00 ± 5,69	0,001**
48th week	74,00 ± 5,83	64,00 ± 5,69	0,001**
52nd week	78,00 ± 5,83	64,00 ± 5,69	0,001**

aStudent’s t-test *p < 0,05 **p < 0,01

### Complications

In the UCBL-only group, 12 patients (% 52, 17) developed pressure sores within the first three months of treatment, resulting in superficial dermabrasion. Six patients had pressure sores both in the medial projection of the talus head and in the lateral malleol, and in six patients only in the medial side. No patient developed superinfection at the wound site. After a week of rest for orthosis treatment and with simple wound dressings, the wound healed, leaving light scar tissue behind. Minor modifications have been made to the parts of the orthosis that put pressure on the foot. No patient had to discontinue treatment. In the group using KT and UCBL, tape-related allergic reactions were observed in only three patients (% 10). In these patients, band therapy was discontinued and treatment with UCBL alone continued. These patients were excluded from the study.

### Radiographic measurements

The results of the right and left TFMA on radiographs of the AP showed no significant difference between the two groups at the pre-treatment, sixth and 12th month measurements (p = 0.544). The right and left TFMA on lateral radiographs again failed to demonstrate a statistically significant difference between measurements taken before treatment and at the six month follow-up (p = 0.544 and p = 0.228, respectively). However, both right and left lateral TFMAs in Group 2 improved from 16,00 ± 5,69 to 14,00 ± 5,69 at the 12th-month follow-up and were found to be significantly different than Group 1 (p = 0.018). The average lateral TFMA was 14.00 ± 5.69 degrees in Group 2 and 10.00 ± 5.83 degrees in Group 1 (Table 3).

**Table 3:** Evaluation of the talocalcaneal angle (TCA), talo-first metatarsal angle (TFMA), calcaneal pitch angle (CPA) and talonavicular angles (TNA)

		Right side				p		Left side				P	
		Group 1 (n = 27) Mean ± SD		Group 2 (n = 23) Mean ± SD				Group1 (n = 27) Mean ± SD		Group2 (n = 23) Mean ± SD			
TC AP	Baseline	42,00 ± 5,83		40,00 ± 5,69		a0,228		42,00 ± 5,83		41,00 ± 5,69		a0,544	
	6th month	38,00 ± 5,83		38,00 ± 5,69		a1,000		38,00 ± 5,83		39,00 ± 5,69		a0,544	
	12th month	35,00 ± 5,83		36,00 ± 5,69		a0,544		35,00 ± 5,83		37,00 ± 5,69		a0,228	
TC LAT	Baseline	32,00 ± 5,83		32,00 ± 5,69		a0,228		32,00 ± 5,83		32,00 ± 5,69		a1,000	
	6th month	30,00 ± 5,83		30,00 ± 5,69		a1,000		28,00 ± 5,83		30,00 ± 5,83		a0,228	
	12th month	24,00 ± 5,83		28,00 ± 5,69		a0,018*		24,00 ± 5,83		28,00 ± 5,69		a0,018*	
TFM AP	Baseline	18,00 ± 5,83		17,00 ± 5,69		a0,544		18,00 ± 5,83		17,00 ± 5,69		a0,544	
	6th month	16,00 ± 5,83		16,00 ± 5,69		a1,000		16,00 ± 5,83		16,00 ± 5,69		a1,000	
	12th month	14,00 ± 5,83		15,00 ± 5,69		a0,544		14,00 ± 5,83		15,00 ± 5,69		a0,544	
TFM LAT	Baseline	15,00 ± 5,83		16,00 ± 5,69		a0,544		15,00 ± 5,83		16,00 ± 5,69		a0,544	
	6th month	13,00 ± 5,83		15,00 ± 5,69		a0,228		13,00 ± 5,83		15,00 ± 5,69		a0,228	
	12th month	10,00 ± 5,83		14,00 ± 5,69		a0,018*		10,00 ± 5,83		14,00 ± 5,69		a0,018*	
CPA LAT	Baseline		11,00 ± 5,83		12,00 ± 5,68		a0,544		11,00 ± 5,83		12,00 ± 5,68		a0,544
	6th month		14,00 ± 5,83		13,00 ± 5,69		a0,544		14,00 ± 5,83		13,00 ± 5,69		a0,544
	12th month		16,00 ± 5,83		15,00 ± 5,69		a0,544		16,00 ± 5,83		15,00 ± 5,69		a0,544
TNA	Baseline		45,00 ± 5,83		45,00 ± 5,69		a1,000		45,00 ± 5,83		45,00 ± 5,69		a1,000
	6th month		43,00 ± 5,83		44,00 ± 5,69		a0,544		43,00 ± 5,83		44,00 ± 5,69		a0,544
	12th month		42,00 ± 5,83		43,00 ± 5,69		a0,544		42,00 ± 5,83		43,00 ± 5,69		a0,544

aStudent-T Test *p < 0,05 **p < 0,01

Pre-treatment, the six month and 12th-month measurements of the lateral CPAs, the AP TCA, the AP TFMA and the TNA of the left and right sides did not have a statistically significant difference between the two groups (p > 0.05). On lateral radiographs, the differences between the right and left TCA results were not statistically significant at the six month follow-up (p = 0.028 and p = 1.000, respectively). However, lateral TCA results of both sides at the 12th-month follow-up, the statistically significant difference in favour of Group 1 was detected during the measurement of the TCA of both sides (p = 0.018). In Group 2, the average lateral TCA was 28.00 ± 5.69 degrees, while the average lateral TCA in Group 1 was 24.00 ± 5.83 degrees (Table 3).

## Discussion

This study attempted to determine if using KT with the UCBL foot orthosis was beneficial to the treatment of SFPP compared to simply wearing the orthosis. Our results suggest that KT is effective in reducing pronation and improving the AOFAS score.

Mereday et al. documented that the UCBL orthosis promotes the correct alignment of the calcaneus [21]. The UCBL orthosis has been demonstrated to have a positive effect on restoring the arch and hindfoot to a normal state for one or more parameters. UCBL can partially restore all of the longitudinal arch's parameters by supporting the bones of the midfoot. In another investigation, Kogler et al. proposed that the contours on the medial surface of the orthosis should support the stabilization of the apical bones of the arch, this would allow the foot to support the longitudinal arches more effectively [22]. UCBL also ensures that the calcaneum is aligned with the tibia in the coronal plane. Clinically, the correct positioning of the calcaneus is considered to be the most significant component of treating flat feet. UCBL also flexes the talus in a dorsal direction, this causes it to stand more erect and approach a normal position. However, it does not have a role in aligning the forefoot. The configuration of the talus is crucial during the movement of the ankle, this is because the talus redistributes the weight to the heel and forefoot. As a result, the head of the talus should be aligned properly. The talus's weight distribution that isn't properly aligned has an abnormal effect. This causes an abnormal amount of stress on the medial calcaneal ligaments and tarsal joints [23].

The talus conveys the weight to the heel and forefoot, which is why the alignment of the talar head during movement of the ankle is crucial. Increased talar inclination causes pronation of the hindfoot, which alters the foot's kinematics. The degree of talar inclination can be gauged with the TFMA, and is associated with a 2.41-fold increase in the likelihood of suffering from symptoms [24–26]. In this study, the use of orthotics had a significant effect on increasing the lateral TFMA in both groups In Group 1, the AOFAS forefoot and midfoot scores were improved by the decrease in talar inclination and improvement in the arch cavus during the natural development of the foot.

**Fig. 2:**
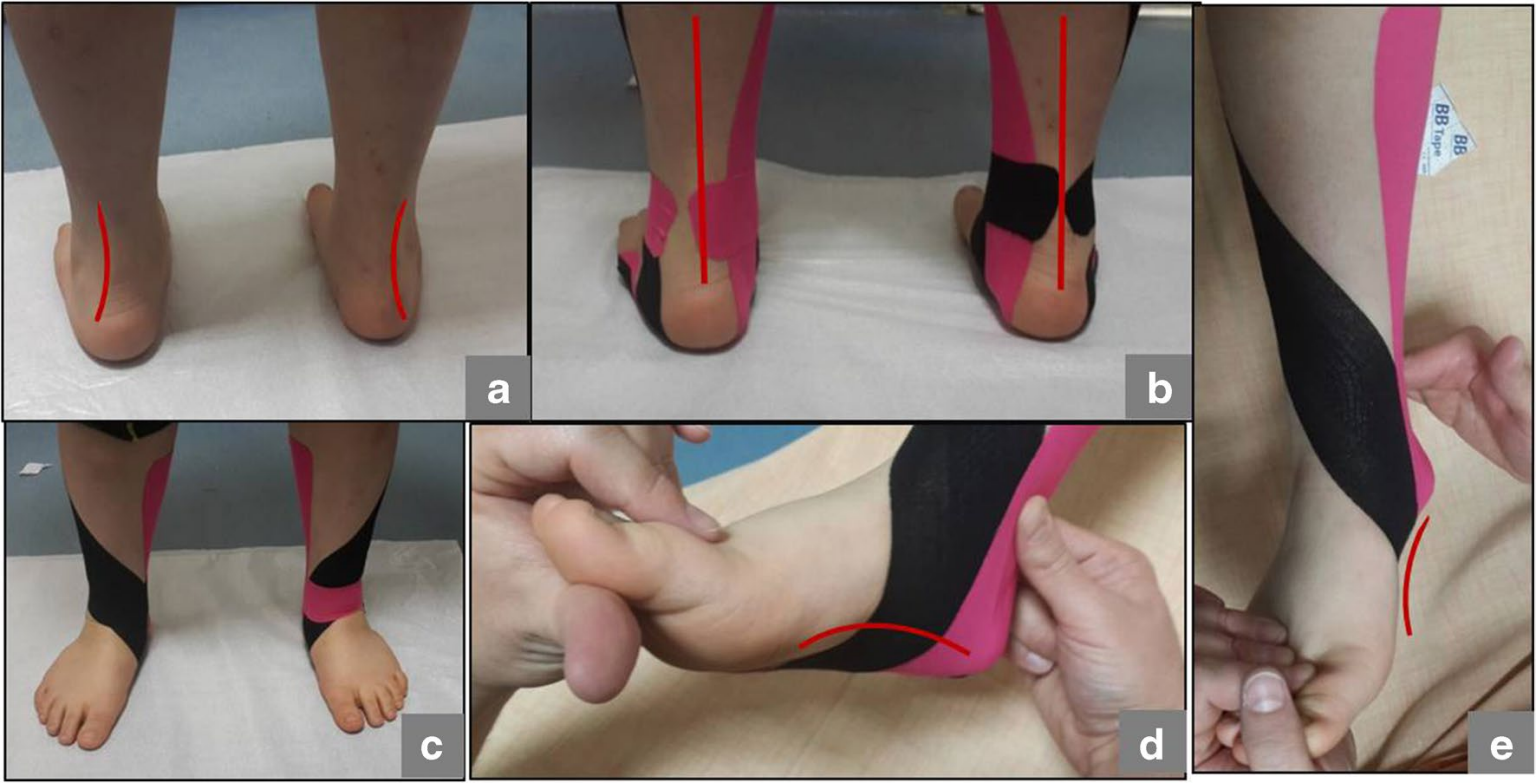
Before (a) and after (b, c, d and e) application of KT. Notice calcaneal alignment (b) and longitudinal arch restoration of the foot (d and e)

**Fig. 3:**
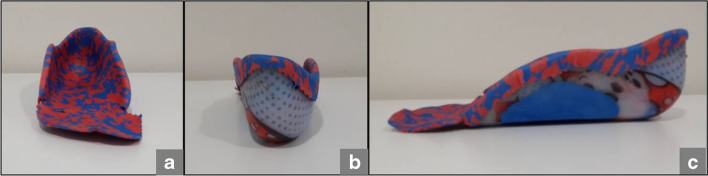
UCBL orthosis. Notice medial support arrives to talar head (a, b and c). Extra support to medial arch to prevent collapse of the medial side (c)

The angle of the calcaneus (CPA) is not consistently associated with the symptoms of flat feet [24]. Additionally, the beneficial effects of orthotic use on the CPA are attributed to the pain alleviation mechanism. Increased CPA after intervention indicates improvement of the deformity; a larger CPA means less plantar flexion of the hindfoot [15]. In this study, the CPA increased significantly following orthosis treatment in both groups. It was observed that the significant increases in both AOFAS scores were accompanied by increases in CPA values (Figs. 2 and 3).

The value of the TCA in diagnosing flatfoot is unclear [27]. The AP TCA is difficult to quantify and inaccurate because of its low correlation to the severity of the disease [27]. Conversely, the effect of orthotic use on the TCA in SFPP patients is significant [26, 28]. The reduction in pain scores has been attributed to the lateral TCA, however, the arched configuration has a poor correlation with pain scores in the SFPP [27, 29]. As a result, the lateral TCA is more effective in evaluating the SFPP. However, Kanatli et al. reported that calcaneal pitch and lateral TCA were not associated with the arch index [9]. With the natural progression of the foot arch, the AOFAS scores for the forefoot and hindfoot tend to increase as a result of other factors, such as an increase in muscle strength and joint flexibility. The CPA and the TCA lateral correlated highly with the AOFAS hindfoot scores. In this study, the use of orthosis had a significant impact on the intertarsal angle in the sagittal plane (TCA and CPA) and helped to alleviate pain by improving the hindfoot alignment and reducing the subtalar subluxation associated with weight-bearing.

The influence of KT on functionality, pain, and movement has been documented in a recent study [12]. The current investigation demonstrated that the addition of additional KT treatment to SFPP had a greater effect than the UCBL-alone treatment. A few theories will be proposed to possibly explain the effectiveness of KT. Additionally, the only significant difference between the groups was the existence of tension created by KT in Group 1 versus Group 2.

The tension of the KT application increases the feedback from the patients to their nervous systems during walking and standing, which increases their balance. KT alters the tactile input, which results in an effect on motor control through a change in the excitability of the central nervous system [30]. Applying tape by pulling in the direction of muscle fibres will promote the contraction of underlying muscles. However, other studies indicated no correlation between tape usage and electromyography-detectable muscle activity or isokinetic dynamometer performance or the tape's effect on the muscle activity was undetectable [31, 32]. The tactile input was sufficient to activate the cutaneous mechanoreceptors that are dedicated to stimulating muscle excitability. However, KT was not powerful enough to produce an increased capacity for muscle power [33].

Increased muscle excitability of the anterior tibia may have contributed to the prevention of excessive pronation and navicular tilt, as a result, the ankle was stabilized in a posteromedial and medial direction [34].

Foot orthotics are commonly used in SFPP. This may lead to several complications. The orthosis is typically rigid or semi-rigid to provide the proper alignment of the tarsal bones; as a result, it increases the pressure on the tarsal bulge, which in turn decreases the likelihood of treatment adherence. The full-stretched KT has the effect of correcting the calcaneal valgus, increasing the arch's height and decreasing the talar head's displacement. Additionally, it may resemble a second skin layer, which would prevent it from developing pressure sores. Furthermore, orthotic shoes are only permitted to be worn in the closed-toe design for a limited period during the day, whereas KT has the advantage of continuous use. In our study, the incidence of pressure sores associated with the use of orthoses is significantly lower when combined with the KT band. The fact that it functions as a second skin layer and applied KT forces cause the foot to maintain its normal contours is the cause of this. The low incidence of pressure sores increases the compliance with the use of orthoses.

Several limitations were present in the current investigation. Initially, the number of participants, their age range and the arch height were not standardized. Second, the AOFAS questionnaire was primarily completed mostly by the parents. The long-term effect of the technique has not been evaluated. Ultimately, our study was non-randomized and retrospective. On the other hand, the fact that the applied KT technique is original and defined by the author first is the strength of the study.

## Conclusion

The findings of our study indicated that KT had a significant effect on postural control. The findings indicated that the implementation of KT with UCBL had a significant impact on the symptoms of pes planus. The KT approach has enhanced the effectiveness of the orthosis, improved the AOFAS scores and prevented complications due to the use of orthosis alone. The necessity of additional research with larger patient populations cannot be denied. Further research may help augmenting empirical evidence regarding the use of KT, and the possibility for its use in preventing deformities and functional inability due to SFPP.

## Data Availability

To access to data and materials, the corresponding author can be contacted.
